# An International Cross-cohort Harmonization and Data Integration Initiative towards Achieving Statistical Power and Meaningful Results

**DOI:** 10.23889/ijpds.v1i1.384

**Published:** 2017-04-19

**Authors:** Tanya Flanagan, Isabel Fortier, Mélanie Fon Sing, Celine Moore

**Affiliations:** 1 Canadian Partnership Against Cancer; 2 Maelstrom Research

## Objectives

The complex interaction between lifestyle, behaviours, genetic factors and the social and physical environment have a fundamental role in modulating risk and/ or progression of health outcomes, especially cancer. To address this complexity, access to large-scale cohorts involving hundreds of thousands of participants and collecting comprehensive and valuable information are required. In the real world however, attaining adequate statistical power presents a major challenge.

Retrospective data harmonization and integration across multiple cohort studies has been shown to be an effective analytical approach to attaining statistical power, with the potential to support population health research and policy related questions and improve our understanding of the complex factors affecting health outcomes. 

## Approach

Large cohorts, with at least 50,000 participants, initiated in countries all over the world, focused on innovative research on cancer and other chronic diseases were invited to participate in this retrospective data harmonization initiative. Cohorts shared their comprehensive metadata related to their study content and design. Almost 150 variables, selected for their relevance to be part of a generic set of information useful for a broad range of research question, were assessed for their harmonization potential and made available on an online searchable study catalogue. Lastly, a proof of concept research question on the retrospective harmonized data was conducted and aimed to investigate methods to analyze individual patient data from multiple studies by studying the determinants associated with age at menopause. 

## Results

Eight cohorts from multiple countries shared their comprehensive metadata related to their study content and design, resulting in over 2 million study participants. Of the 150 potential variables, the majority of them were harmonizable for co-analysis. The proof of concept research question, applied to these variables generated interesting results, widely supported by other research on this topic, found in the literature. This work demonstrates the value of retrospective data harmonization and integration to be an effective analytical approach to attaining statistical power.

The searchable study catalogue, available online for researchers to use in their own international research projects offers a new innovative tool for potential co-analysis of similar measures collected by separate cohort studies. 

## Conclusions

Retrospective harmonization offers an innovative approach to optimize use of existing research data with increased statistical power.

